# Reply to: Severe hypercalcemia as a form of acute lymphoblastic leukemia presentation in children

**DOI:** 10.5935/0103-507X.20160035

**Published:** 2016

**Authors:** Andreia Luís Martins, Marta Moniz, Pedro Sampaio Nunes, Clara Abadesso, Helena Cristina Loureiro, Helena Isabel Almeida, Ximo Duarte

**Affiliations:** 1 Pediatric Intensive Care Unit, Hospital Prof. Doutor Fernando Fonseca, EPE - Amadora, Portugal.; 2 Oncology Department of Children and Adolescents, Instituto Português de Oncologia Lisboa, Francisco Gentil, EPE - Lisboa, Portugal.

We appreciate your interest in our article.

Hypercalcemia of malignancy in children is in fact well described; however, this complication is rare and corresponds to 0.4% - 1.3% of cases of cancer in children.^(1,2)^ The risk of fatal outcomes of severe hypercalcemia either due to cardiac or neurological complications is also well known. These severe cases require aggressive treatment involving volume expansion and treatment with calcitonin and bisphosphonates.^(3)^ In the case described, which involved severe hypercalcemia and deterioration of the state of consciousness, we opted for hyper-hydration and treatment with zoledronate and hemodiafiltration.^(4)^ The zoledronate, rather than pamidronate, was preferred because of its greater potency and efficacy and shorter period of treatment, as previously described.^(5)^ The decision to initiate hemodiafiltration involved the consideration that calcitonin is not marketed in Portugal and that the maximum effect of bisphosphonates is not observed before two to four days after administration.^(5)^ Therefore, the urgent need to reverse the ionic imbalance in a child with a Glasgow coma score of 8 led to the performance of hemodiafiltration. It has been reported that parathyroid hormone related peptide (PTHrp) is an important mediator in hypercalcemia of malignancy and is the mediator most often associated with this malignancy,^(2,6)^ which explains its importance in the differential diagnosis of hypercalcemia. However, in this case, the dose of PTHrp was 1.2pmol/L (reference value < 2.0), as shown in table 2.^(4)^ As reported in Martins et al.,^(4)^ two mechanisms are responsible for malignant hypercalcemia: (1) local osteolytic lesion (bone metastases) and (2) humoral hypercalcemia via activation of the receptor activator of nuclear factor kappa B/receptor activator of nuclear factor kappa B ligand (RANK-RANKL). Although PTHrp is the mediator most often identified, other mediators may be involved, including interleukin (IL)-1, IL-6, tumor necrosis factor (TNF-α), transformation growth factor beta (TGF-β), prostaglandins, calcitriol, and PTH that is produced ectopically.^(6)^ In addition, the medium- and long-term adverse effects of bisphosphonates have been associated with osteonecrosis of the jaw and ectopic deposition of calcium.^(7)^ However, to date, no adverse effects related to bisphosphonates have been observed in the described case. Nevertheless, hypocalcemia occurred for 10 days after the initiation of treatment with zoledronate, highlighting the need to monitor serum calcium levels two to four weeks after the initiation of treatment, which is the period of activity of bisphosphonates.

Andreia Luís Martins, Marta Moniz, Pedro Sampaio Nunes, Clara Abadesso, Helena Cristina Loureiro and Helena Isabel Almeida

Pediatric Intensive Care Unit, Hospital Prof. Doutor Fernando Fonseca, EPE - Amadora, Portugal.

Ximo Duarte

Oncology Department of Children and Adolescents, Instituto Português de Oncologia Lisboa, Francisco Gentil, EPE - Lisboa, Portugal.
